# Situational Analysis of Healthcare Delivery and User Perspectives of Mobile Diagnostics (mDiagnostics) in Chennai, Tamil Nadu, India: A Mixed-Method Study

**DOI:** 10.7759/cureus.45808

**Published:** 2023-09-23

**Authors:** Vinoth Gnana Chellaiyan Devanbu, Narendranath R, Sanjutha A, Neeta Kumar

**Affiliations:** 1 Community Medicine, Chettinad Hospital and Research Institute, Chettinad Academy of Research and Education, Chennai, IND; 2 Community Medicine, KMCH Institute of Health Sciences and Research, Coimbatore, IND; 3 Social Health Implementation, Indian Council of Medical Research (ICMR), New Delhi, IND

**Keywords:** mdiagnostics, labike, affordability, healthcare facility, situational analysis

## Abstract

Introduction

Situational analysis of exciting infrastructure including mobile health services is crucial for comprehensive healthcare delivery. The concept of mDiagnostics has gained traction as it addresses the challenges of accessibility, affordability, and availability of healthcare services in remote regions.

Purpose

The study was to do a situational analysis of the availability of medical diagnostic facilities and identify the challenges and barriers faced in the implementation and utilisation of mDiagnostics.

Material and methods

The present study was a mixed mixed-method study conducted in rural and urban areas of Chennai, Tamil Nadu, India. A total of 1,489 households were included. Situational analysis of existing healthcare facilities and the availability of Ayushman Bharat Health Account (ABHA) numbers for study participants in both urban and rural areas was assessed. In-depth interviews on user perspective, affordability, awareness of existing health services, and perception of the utility of mobile lab services and focus group discussions with healthcare professionals, community members, and key stakeholders were carried out. Thematic analysis for qualitative data, proportion, and means were calculated for the quantitative component.

Result

Out of 1,489 households included, 711 were from rural areas, and 778 were from urban areas. The distance traveled from their residence to both the lab and health facility was less than 5 km in urban areas, while it is more than 5 km in rural areas. The mean expenditure in availing healthcare services is above five thousand rupees per annum in nearly half of the rural households (46%) and 60% of urban. The analyses of interviews explored the availability, acceptability, and affordability under seven thematic areas for situational analysis of healthcare facilities, and a focused group discussion was held to explore the community member’s barrier to healthcare services.

Conclusion

The study reveals a comprehensive understanding of healthcare delivery access disparities between rural and urban areas in Tamil Nadu. The findings highlighted the potential benefits of mobile lab initiatives in improving healthcare access and early disease detection in underserved rural communities.

## Introduction

India is a country with a huge population, and with that, the healthcare problem in India is also vast. India spends almost 2.1% of their GDP on healthcare [1], but still, there remain many differences in the quality of care between rural and urban areas, as well as between public and private healthcare systems. India's competitive advantage lies in its large pool of well-trained medical professionals. India is also cost-competitive compared to its peers in Asia and Western countries [1]. The gap between rural and urban healthcare systems is large, where, in rural areas, there is a shortage of proper medical facilities such as medicine, doctors, etc. Additionally, disparities between private and public healthcare systems exist [2]. The public healthcare system lacks in manifold, such as deficient infrastructure, deficit manpower, equivocal quality of services, etc. While the condition of private health services in India is good and provides good facilities to patients, we have expensive health services for healthcare delivery [3]. The National Health Authority of India has been implementing Pradhan Mantri Jan Arogya Yojana (PM-JAY) or “Ayushman Bharat” [4] aiming at providing quality healthcare to all citizens at an affordable rate by reducing out-of-pocket expenditures (OOPEs) through cashless services. 

The Indian rural communities face numerous challenges in accessing quality healthcare services, primarily due to limited infrastructure, geographical barriers, and a shortage of trained healthcare professionals. The healthcare disparities between rural and urban areas have been a longstanding concern, and efforts to bridge this gap have been a priority for policymakers and public health experts. In recent years, the introduction of mobile health services especially lab facilities has emerged as a potential solution to improve healthcare access and delivery in rural India [5].

mDiagnostics involves the deployment of mobile healthcare units to remote and underserved rural areas. These units are equipped with essential medical equipment and staffed with trained healthcare professionals who offer diagnostic testing, health screenings, and basic medical consultations to the local population. The concept of mobile healthcare units has gained traction as it addresses the challenges of accessibility, affordability, and availability of healthcare services in remote regions [6]. Despite the growing importance of mDiagnostics in rural healthcare delivery, there is a notable lack of comprehensive research exploring their effectiveness, impact, and potential areas for improvement. A qualitative research study is essential to provide deeper insights into the experiences, perceptions, and challenges faced by both healthcare providers and rural community members in the implementation and utilization of mDiagnostics. By exploring the perceptions and experiences of rural community members who have utilized mobile lab services for healthcare access, the study seeks to understand the effectiveness and reach of these services from the beneficiaries' standpoint. There is a need to identify the challenges and barriers faced by healthcare providers and community members in the implementation and utilization of mDiagnostics. The present study is a mixed-method research study that aims to address this knowledge gap by investigating the experiences and perspectives of healthcare providers involved in delivering mDiagnostics in rural India. This research will assess the impact of mDiagnostics on healthcare access and health outcomes in rural communities. Understanding the potential benefits and limitations of these services in early detection, managing chronic conditions, and promoting preventive care will be crucial in devising strategies to enhance rural healthcare delivery [7,8].

## Materials and methods

Study design

The study design was a mixed-method study design.

Study setting

The present study was conducted in urban and rural areas of Chennai, Tamil Nadu, India. A list of urban and rural areas of the selected district was prepared, and one rural and one urban site was selected randomly. In-depth interviews and focus group discussions were conducted at the selected rural site.

Study population

The study population in the study for the quantitative part was the head of the household. The head of the household was contacted to obtain consent. For the qualitative component, in-depth interviews and focus group discussions were done. For in-depth interviews, healthcare professionals, community members, and key stakeholders involved in mobile lab services were purposefully selected to participate in the study. For focus group discussion, community members of the rural area were randomly selected.

Sample size

For the quantitative component, all the households in the selected urban and rural areas were included in the study. In the selected rural and urban study sites, 711 rural and 778 urban households were included.

For the qualitative component, in-depth interviews, a total of 14 persons were interviewed, including healthcare professionals, community members, and key stakeholders involved in mobile lab services, which were randomly selected, and interviews were conducted till the point of saturation. For focus group discussions, eight community members of the rural area were randomly selected, and the discussion was conducted till the point of saturation.

Study period

The study was carried out over a period of one year from June 2022 to May 2023.

Study tool

The study tool used was a questionnaire for the quantitative component.

Quantitative Component

Baseline details, including the education, and occupation of the head of the household were collected. Health-seeking behavior and details of existing healthcare facilities were collected. Availability of Ayushman Bharat Health Account (ABHA) number and reasons for not having if not available were registered. An ABHA number is a 14-digit unique number used to identify and authenticate people to thread their health records across multiple healthcare service providers.

Qualitative Component

Semi-structured in-dept interviews were conducted with healthcare providers (medical officers-doctors, nursing staff, and lab technicians), community members, and stakeholders to explore their experiences, perceptions, and challenges related to mobile lab services. Interviews were audio-recorded and transcribed for analysis. Focus group discussions were conducted with community members to encourage open discussions and gather collective insights.

Study methodology

A mixed-method explanatory study using purposive sampling was conducted in the rural and urban populations of Chennai. A questionnaire was used to collect information such as baseline details of education, occupation of the head of the household health-seeking behavior, and details of healthcare facilities and the availability of ABHA numbers in both urban and rural areas. In-depth interviews and focus group discussions with healthcare professionals, community members, and key stakeholders involved in the mDiagnostic initiative.

Data analysis

Data entry and analysis for quantitative data in Microsoft Excel spreadsheet and subsequently analyzed with Statistical Product and Service Solutions (SPSS, version 21.0) (SPSS Statistics for Windows, Armonk, NY).

Quantitative Study

Proportions and means were calculated. A chi-square test was conducted.

Qualitative Study

The collected data were analyzed using thematic analysis. Transcripts from interviews and focus group discussions were coded and categorized into themes and sub-themes. NVivo software (NVivo version 14.23.0, QSR International) was used in managing and analyzing the qualitative data. Triangulation of data from multiple sources enhanced the rigor and validity of the findings.

Thematic analysis

The transcribed data were analyzed using thematic analysis. Themes and sub-themes were identified based on recurrent patterns in the data. The service users were interviewed in-depth on the seven thematic areas for situational analysis of healthcare facilities - affordability, healthcare quality in public and private hospitals, availability of advanced facilities, acceptability of indigenous system of medicine, distance of healthcare facilities, and awareness of health and health-related states. Barriers were assessed by structural, financial, and cognitive domains. A focused group discussion was held to understand the barriers to the healthcare system in the community. All the questions and discussions were held in the regional languages of the site. The total duration was 180 minutes, with the session for the situational analysis of 140 minutes (20 minutes for each theme) and 40 minutes for barriers. The themes for the qualitative study of community-level acceptability of technology in health included affordability, healthcare quality in public hospitals, healthcare quality in private hospitals, availability of advanced facilities, acceptability of indigenous system of medicine, distance of healthcare facilities, and awareness of health and health-related states. Barriers were assessed - structural, financial, and cognitive domains.

1. Data triangulation: Triangulation was used to validate the findings, comparing data from different sources and perspectives.

2. Member checking: Researchers shared the findings with participants to validate the accuracy and interpretation of the data.

3. Peer review: External experts were invited to review the research design and findings for added validity.

Ethical Consideration

The study was conducted after getting Ethics Committee approval. Informed consent was obtained from the study participants, and confidentiality was ensured.

## Results

Quantitative study finding

Out of 1,489 households included, 711 were from rural areas, and 778 were from urban areas. The mean age of the participants of the respondents was 41.4±13.74 years. Nearly 99% of people living in urban areas do not have an ABHA number for at least one person in the family. However, only 4% of people in rural households have ABHA numbers, and the most common reason for non-availability was unawareness about it in both urban and rural areas. Moreover, the literacy rate was found to be equal in rural and urban areas; 52% of participants in rural and 40% of participants in urban were semi-skilled workers (Table 1).

**Table 1:** Health condition and health-seeking behavior patterns among rural and urban households (n=1,489).

Variables		Rural	Urban	p-value
		n (%)	n (%)	
Disease history	Communicable diseases (bacterial infections)	586 (82.1)	643 (85)	0.171
	Communicable diseases (viral infections)	30 (4.2)	34 (4.2)	
	Malignancies	57 (8)	74 (9.3)	
	Hormonal disorders	7 (0.9)	3 (0.3)	
	Noncommunicable disease	21 (3)	16 (2)	
	Nutritional disorders	7 (2.7)	6 (0.5)	
Reason for visiting a doctor	Cost-effective	98 (14)	177 (22.5)	0.000
	A doctor is ready to help	20 (2.8)	152 (20.5)	
	Fear of disease	591 (83)	0 (0)	
	Service available in close proximity	17 (1.2)	445 (57)	
Distance traveled for lab testing	1-5 km	3 (0.4)	759 (97.6)	0.000
	More than 5 km	708 (99.6)	19 (2.4)	
Doctor preference	Government doctor	191 (27)	239 (30.5)	0.000
	Private doctor	74 (10)	4 (0.5)	
	Both	446 (63)	535 (69)	
Distance to health facility from residence	1-5 km	296 (42)	778 (100)	0.000
	6-10 km	213 (30)	0 (0)	
	11-15 km	15 (28)	0 (0)	
Know about mobile health applications	No	704 (99)	760 (97.7)	0.046
	Yes	7 (1)	18 (2.3)	
Self-writing health diary	No	155 (22)	81 (10)	0.000
	Yes	556 (78)	697 (90)	
Reason for not having a health diary	No awareness	685 (96.3)	769 (98.8)	0.000
	Illiterate	0 (0)	9 (1.2)	
	Not interested	24 (3.3)	0 (0)	
	Not skilled	1 (0.4)	0 (0)	
Treatment cost (per annum)	0-1000	158 (22.2)	349 (39)	0.000
	1000-5000	325 (46)	412 (60)	
	5000-10000	13 (1.8)	8 (1)	
	10000-15000	6 (1)	0 (0)	
Travel cost (per annum)	0-500	340 (48)	683 (90)	0.000
	500-1,000	142 (20)	78 (10)	
	1,000-1,500	12 (2)	0 (0)	
	1,500-2,000	6 (1)	0 (0)	
Have a unique health ID	No	705 (99)	777 (99.9)	0.044
	Yes	6 (1)	1 (0.1)	

Health Status and Health-Seeking Behavior

The health status of the households revealed that the most common illness was communicable disease, and the prevalence was almost the same among rural and urban households, with the predominance of bacterial infections at 85% in urban and at 82% in rural, followed by malignancies. Both urban and rural prefer both government and private doctors for healthcare. The distance traveled from their residence to both the lab and health facility was less than 5 km in urban areas, while it was more than 5 km in rural areas. Nearly half of the rural households (46% and 60% of urban households) spend up to five thousand rupees for their treatment in health services per annum. A significant difference between rural and urban existed in the reason for visiting a doctor, distance traveled for testing and health facilities, and the reason for having and not having self-diary, travel, and treatment costs among urban and rural households (Table 2).

**Table 2:** Socio-demographic characteristics of urban and rural households (n=1,489).

Variables		Rural n (%)	Urban n (%)	p-value
Have ABHA numbers for family members	No	681 (96)	777 (99)	0.000
	Yes	30 (4)	1 (1)	
Reason for not having ABHA number	Not aware of it	510 (72)	352 (45)	0.000
	Enrollment not done	110 (15)	92 (12)	
	Yet to receive a number	9 1(13)	334 (43)	
Education	Postgraduate	10 (1.4)	9 (1.1)	0.003
	Graduate	60 (8.4)	40 (5.1)	
	Higher secondary	124 (17.4)	99 (12.8)	
	Secondary	99 (14)	117 (15)	
	Middle	102 (14.3)	109 (14)	
	Primary	146 (20.5)	216 (28)	
	Illiterate	170 (24)	188 (24)	
Occupation	Professional	1 (0.1)	5 (0.6)	0.000
	Semi-professional	12 (1.6)	82 (10.1)	
	Skilled	134 (19)	207 (27)	
	Semi-skilled	370 (52)	305 (39.2)	
	Unskilled	192 (27)	178 (23)	
	Unemployed	2 (0.3)	1 (0.1)	
No. of family members	Up to 5	667 (94)	741 (95)	0.135
	>5	44 (6)	37 (5)	
House ownership	Own	702 (99)	588 (75.5)	0.000

Qualitative study findings

Community User Perspective of Health Services

Affordability - Grouped under the cost of preventive services - screening and vaccination, cost of outpatient services, cost of inpatient services, and cost of advanced care.

“Of course, we have financial challenges when utilizing healthcare services. The only way to secure funding for treatment is to borrow money, and occasionally sell our goats or hens.”

(A 44-year-old female)

"Access to healthcare services was also said to be hampered by direct expenses connected to assuring "good treatment" in private institutions. Since we are farmers, money is not always readily available. Ten kilometers away, there is a private clinic with user costs between 1,500 and 2,000 rupees. Therefore, despite our desire to visit, we are unable to do so."

(A 38-year-old male)

"The majority of participants recognized that having non-communicable diseases (NCDs) or any other chronic illness adds to their household's financial burden. Therefore, whether or not they were cured, people with severe illnesses may discontinue their therapy midway owing to financial worries. I haven't had a serious illness in ten years. I've already sold half of my property to pay for medical costs. I have stopped seeking treatment because of this."

(A 49-year-old male)

"My joints have been hurting for the past two years. I had attended Chengalpet Medical College twice. After that, though, I no longer receive therapy due to cost due to the fact that my spouse is a small farmer."

(A 44-year-old female)

Healthcare quality in public hospitals - standards of healthcare delivery by health personnel, waiting time, availability of adequate manpower, and health facility working hours.

“A 32-year-old husband brought his expectant wife to a neighborhood primary health center for a check-up, but they were unable to see a doctor because of the long queue. They eventually had to go over 12 kilometers from their home to a private clinic in a neighboring town, where there were no good roads or other means of connection. I brought my wife in for a checkup, but despite coming in repeatedly for three days, I was unable to even see the doctor. At the counter, I asked someone I knew to make a reservation, but even that didn't work.”

(A 28-year-old male)

“Most of the time, doctors are not available, and even in the most urgent situations, we are unable to find one.”

(A 35-year-old female)

Availability of advanced facilities - diagnostic services - laboratory, diagnostic services - imaging, referral services - ambulance, linked tertiary care hospital, and use of technology in health.

The respondents discussed rudeness/behavior on the part of medical professionals, particularly if the patient was from a low-income family.

"The 37-year-old woman, Kanniammal, was frail. Her spouse worked as a landless farmer. She didn't anticipate being disrespected. “After examining me, the doctor informed me that I would not be able to afford therapy. I was so hurt and defeated that I never had the guts to go back to the doctor.”

“Only cold, cough, and fever medications are offered in the neighborhood pharmacy. It usually takes 3-4 days for fresh medications to arrive when the current supply of prescriptions runs out.”

(A 44-year-old male)

The participants claimed that inadequate supplies and equipment at the government health facilities resulted in subpar treatment. People complained about the absence of equipment, particularly in diagnostic services.

“This hospital does not have a facility for the treatment of serious, significant illnesses. Even for a CT scan or ultrasound, we need to go quite a distance to a nearby town. If all diseases had been treated locally, it would have benefited those like us who are poor.”

(A 34-year-old female)

Acceptability of indigenous system of medicine - knowledge of the availability of services, the practice of traditional methods at the village level, and perception of allopathic medicine and AYUSH.

“Since I am accustomed to traditional medicine, I feel soothed when I utilize it. I have more confidence in conventional treatments.”

(A 36-year-old male)

“We are accustomed to utilizing conventional medications because we have done so since birth. Additionally, they can occasionally be more beneficial than modern ones.”

(A 34-year-old female)

The Meesaikar provide medical care at substantially lower costs, making them widely accessible to individuals who are primarily low-wage agricultural laborers.

"For most ailments, such as jaundice, fever, cough, cold, chickenpox, and pneumonia, traditional remedies may be acquired for around Rs 10-20; therefore, we tend to opt for them more frequently."

“Even the price of the medications is negotiable, and we are free to buy them in whatever amount.”

(A 42-year-old male)

Distance of healthcare facility - distance from home - first-level health facility, distance from the workplace - first-level health facility, and distance of referral hospital.

The 33-year-old mother of a four-year-old, whose name was Ramesh Krishna, was a respondent. While playing, her kid suffered a bone fracture in his left leg. She took her son to a traditional healer who is known for puttur bandage after hearing advice from others, who strapped his hand with medicinal herbs and told the mother she did not need to take her son to a doctor because he would soon be well.

She took him to a private doctor after 15 days, who indicated they had brought him too late because the hand had been incorrectly wrapped up. She was informed that surgery would be required to correct the boy's deformities.

Awareness of health and health-related states - health awareness of common communicable diseases, health awareness of noncommunicable diseases, and awareness of beneficiary programs.

“The fact that the healthcare facilities are at least 10 to 15 kilometres away from our village makes visiting them a significant challenge. Additionally, our location lacks any public transportation. We are unable to continue walking, therefore our only option is to arrange a vehicle on our own.”

(A 38-year-old male)

The Facility of Mdiagnostics

 “Mobile health services such as lab facilities and healthcare delivery will be useful to the community as everyone in the village can avail the facilities without much traveling.”

(A 46-year-old male)

“Mobile lab facilities and availability of reports help us to get ourselves tested at the community itself without having to travel and incur our loss of daily wages.”

(A 24-year-old female)

“Mobile health services and mobile lab facilities are boons to people like who can't walk to get public transport to reach nearby health facility.”

(A 74-year-old female)

Provider’s Perspective of Health Services

Understaffed health centers: The healthcare professionals claimed that a shortage of human resources caused them to be overworked by the volume of patients.

“There is not enough staff in this model hospital. There are only three doctors and two GNM nurses in this facility. Every day, more than 100 patients arrive, making it impossible for us to spend adequate time with each one."

(A 38-year-old male doctor)

Low literacy and poor compliance: “In the past ten months, I have observed and experienced that most patients seek out conventional treatments first and then turn to us if such treatments are unsuccessful in curing them. It was thought that people with low levels of education had misconceptions regarding specific medications and testing.”

(A 30-year-old male doctor)

“Pregnant women who are given iron supplements do not take them. They believe it will make the baby heavier. Some claim that taking those medications causes them to smell a certain way.”

(A 35-year-old ANM nurse at a sub-center)

“The patients are uneducated, thus they find it difficult to understand what we say. People are reluctant to come here. Even when individuals are educated, they are not wise.”

(A 42-year-old female doctor at PHC)

Connectivity and poor communication: “To treat pregnant ladies and administer vaccinations, we must go door to door. Because of the flooding during the monsoon season, it is difficult to visit the homes and provide care. The inadequate road system makes it very difficult for us to carry out our tasks.”

(A 40-year-old ANM sub-center nurse)

“The area's distances were another issue. The public health facilities were at least 10-15 kilometers away from the settlements. Lack of public transport made the issue worse.”

“Due to the distance, a patient who experiences an emergency at night will not travel to the hospital. He or she will hold off until the next day. Additionally, they struggle to arrange a vehicle at night.”

## Discussion

India is rapidly undergoing an epidemiological transition with a sudden change in the disease profile of its population. A higher prevalence of self-reported morbidity was observed among the elderly and females, particularly in the urban locality. On the contrary, the rural population, due to their sociocultural taboo being deeply rooted in their society [9], was prevented from benefiting from quality health services. Adding on to the constraints, it faces severe challenges due to the doubling burden of communicable and noncommunicable diseases. A practical, innovative, and responsive public health system needs to be planned to make healthcare services available for NCDs and CDs at the primary level for both urban and rural areas. In order to ameliorate caregiving, the involvement of the community is critical [10].

There is a constant OOPEs for healthcare that has been a burden irrespective of their dwelling. According to our study, nearly half of the rural households (46%) and 60% of urban households spend up to five thousand rupees for their treatment in health services per annum. To minimize the burden of OOPEs, Ayushman Bharat Health provides health insurance coverage to Indian citizens who are below the poverty line.

Adding to the above, inadequate infrastructure, restricted access to medical care, lack of doctors and other healthcare personnel, and a lack of adequate health budgeting are a few of the difficulties that the healthcare delivery system must overcome in India. Innovative solutions that can increase access to healthcare in rural India are required to address these issues.

Our study’s situational analysis of the availability of medical diagnostic and testing facilities in underprivileged/remote areas acknowledged that accessibility in terms of distance is a detrimental factor. With all such credo came the doorstep healthcare services through the Labike initiative, which has been initiated to decrease the travel burden of the rural and urban population.

Our qualitative data findings shed light on the advantages and limitations of mDiagnostics on disease early detection, treatment initiation, and overall health in rural India. Both service users and providers identified that health service utilization in the study area was affected by the acceptability of Technology and certain other barriers. Based on the participant’s statements Healthcare providers reported a sense of fulfillment in serving rural communities through mobile lab services. The vitality of the healthcare providers is an important factor for the success of any new initiative. According to a study conducted by Zamani-Alavijeh et al. [11], self-efficacy means the educator’s faith in his/her own abilities to hold educational programs and sessions, which were considered an important factor in their success and in performing their duties. In a study conducted by Bandura [12], the participants emphasized the key role of self-efficacy in creating professional feelings and using its power. The providers did mention that training and support were highlighted as essential factors in ensuring effective service delivery. It was suggested [13] that training these informal providers may be one way to improve the quality of care where few alternatives exist.

Even though the providers reported fulfillment, certain challenges related to limited resources, a lack of proper infrastructure, and difficulty in reaching remote areas were concerns. According to Singh [14], the government succeeded in generating infrastructure in urban areas but failed to do so in rural, sustaining 70% of the Indian population. According to Ballard [15], money that the government spends is largely distributed to urban settings, rather than rural ones. Moreover, the private healthcare industry primarily serves urban settings. This significant shortage is throughout India but is particularly problematic in rural areas. Rural community members acknowledged the convenience and accessibility of mobile lab services in their locality. This initiative overcomes the burden of difficulty in transporting equipment. Particularly, in rural areas, “Labike” offers a solution for overcoming barriers of limited access to diagnostic tests for early detection [16].

Nevertheless, the boon has a few concerns; according to Singh [14], only 17% of health expenditure is borne by the state, with 82% out-of-pocket expenditure, making it the sixth lowest country in the world for public health spending. The key operating principles for sustainable healthcare ventures at the base of the pyramid focus on the four A's: accessible, affordable, acceptable, and awareness [17,18-25]. Insufficient government investments in healthcare severely constrain health services access at the rural bottom of the pyramid. This study helped us explore the perceptions of healthcare providers and utilizers, the challenges and barriers of implementation, and the impact of mDiagnostics. Improved health awareness and preventive care practices were observed in communities, and community members expressed a heightened sense of trust and confidence in the healthcare system due to the presence of mobile labs.

Though all issues could not be addressed with this limited study population, there are a few limitations to the study. This qualitative study neither could quantify OOPE for further follow-up of management nor improve their logistic challenges.

## Conclusions

In conclusion, the quantitative study reveals a comprehensive understanding of healthcare delivery access disparities between rural and urban areas in Tamil Nadu. The findings emphasize the pressing need for targeted interventions and policy reforms to bridge this gap and ensure equitable healthcare services for all residents across the region. This study gave rise to a newer innovative, mDiagnostics initiative “Labike” to succor the impoverished. The qualitative study on mDiagnostics in rural India provided valuable insights into the experiences, challenges, and impact of these services. The findings highlighted the potential benefits of mobile lab initiatives in improving healthcare access and early disease detection in underserved rural communities. Moreover, the study emphasized the importance of addressing logistical challenges and ensuring long-term sustainability to maximize the impact of mDiagnostics in rural India.

The findings underscore the need for targeted interventions that address the unique challenges faced by these communities, fostering equitable access to quality healthcare services and technological advancements. As healthcare continues to evolve, the insights from this study can guide policymakers, healthcare practitioners, and technology developers in devising effective strategies that improve the health outcomes for all residents of Tamil Nadu.
